# Leprosy

**Published:** 1902-06-07

**Authors:** 


### LEPROSY.

At the last meeting of the Royal Medical and Chirurgical Society two communications on the subject of leprosy were read, the discussion upon which was adjourned to the next meeting on June 10th. Mr. T. J. Tonkin described leprosy as it is met with in the Soudan. In regard to etiology he had satisfied himself that the disease was communicated by a process of mediate infection in which clothes played the chief part. In the Soudan, he said, clothes were hardly ever washed. Rich men bought their clothes new, and when they were soiled sold or gave them away, so that in the course of its existence a robe might have had an almost unlimited number of owners, any one of whom might have suffered from leprosy. Moreover, natives did not object to wear robes which had been worn by lepers, even when stiff with exudation, and Mr. Tonkin attributed the infection to the rubbing of these robes between the prominences of the body and the hard matted surfaces which served the natives as beds, for, he said, it was exactly upon these prominences that (in the Soudan at least) the first signs of the disease usually appeared. As to predisposing causes he held that in every leper field in the world the diet was defective in nitrogenous constituents. Mr. Jonathan Hutchinson then made a communication on leprosy in Cape Colony and Natal, based upon his experiences during his recent tour. While maintaining the fish theory he said that in the Kaffir kraals of Natal there was good reason to believe that the disease was communicated from person to person, and especially to young children, and he suggested that the mode of communication was by eating food direct from a leper's hands, contaminated by secretions containing the living bacillus. This he proposed to call "commensal communication." It seemed to him probable that in Cape Colony *de novo* production—*i.e.*, without hereditary transmission or direct infection—by the use of salt fish, had been the common cause of the disease, whilst in Natal commensal communication had been the more frequent. The chief object of his paper was to maintain two principal propositions: first, that leprosy was undoubtedly communicable from person to person, but that the mode of communication was peculiar and did not come under the head of contagion properly so called; second, that the facts as regards the origin and distribution of leprosy in

South Africa strongly favoured the belief that it could arise without personal contagion as a specialised form of disease, possibly tuberculous, arising from the eating of imperfectly cured fish. The first symptoms of leprosy were, he said, almost always those of a blood disease. There was never any primary sore or other indication of local infection. The earliest phenomena were as a rule bilateral, and thus implied blood contamination, and the suggestion that the bacillus was received into the stomach, by the direct contamination of food by leprous discharges, met the conditions of the case. The discussion raised by two papers maintaining such directly opposite conclusions ought to be interesting.

